# Optimal peritoneal cancer index cutoff point for predicting surgical resectability of pseudomyxoma peritonei in treatment-naive patients

**DOI:** 10.1186/s12957-024-03318-4

**Published:** 2024-01-31

**Authors:** Mingjian Bai, Yunxiang Li, Hairong Pu, Yueming Xu, Jingliang Chen, Hongbin Xu, Hongjiang Wei, Guowei Liang, Ruiqing Ma, Jing Feng

**Affiliations:** 1 Department of Clinical Laboratory, Aerospace Center Hospital, 15 Yuquan Road, Haidian District, Beijing, 100049 China; 2 Institute of Genetics and Department of Human Genetics, Zhejiang University School of Medicine, Hangzhou, 310058 China; 3 Department of Literature and Science, University of Wisconsin-Madison, Madison, WI 50155 USA; 4 Department of Myxoma, Aerospace Center Hospital, Beijing, 100049 China; 5 Department of Radiology, Aerospace Center Hospital, Beijing, 100049 China

**Keywords:** Peritoneal cancer index, Cutoff point, Surgery, Pseudomyxoma peritonei

## Abstract

**Background:**

The peritoneal cancer index (PCI) has been used to predict surgical outcomes for pseudomyxoma peritonei (PMP). The present study aimed to establish the optimal cutoff point for PCI to predict surgical resectability of PMP.

**Methods:**

A total of 366 PMP patients were included. The patients were divided into low-grade and high-grade groups. Based on the completeness of the cytoreduction (CC) score, both low-grade and high-grade PMP patients were further divided into complete cytoreductive surgery (CRS) and maximal tumor debulking (MTD) subgroups. The ability to predict surgical resectability of total and selected PCI (regions 2 + 9 to 12) was analyzed through receiver operating characteristic (ROC) curves.

**Results:**

Both total and selected PCI demonstrated excellent discriminative ability in predicting surgical resectability for low-grade PMP patients (*n* = 266), with the ROC-AUC of 0.940 (95% *CI*: 0.904–0.965) and 0.927 (95% *CI*: 0.889–0.955). The corresponding optimal cutoff point was 21 and 5, respectively. For high-grade PMP patients (*n* = 100), both total and selected PCI exhibited good performance in predicting surgical resectability, with the ROC-AUC of 0.894 (95% *CI*: 0.816–0.946) and 0.888 (95% *CI*: 0.810–0.943); correspondingly, the optimal cutoff point was 25 and 8, respectively. The discriminative ability between total and selected PCI in predicting surgical resectability did not show a statistical difference.

**Conclusions:**

Both total and selected PCI exhibited good performance and similarity in predicting complete surgical resection for both low-grade and high-grade PMP patients. However, the selected PCI was simpler and time-saving in clinical practice. In the future, new imaging techniques or predictive models may be developed to better predict PCI preoperatively, which might assist in confirming whether complete surgical resection can be achieved.

## Introduction

Pseudomyxoma peritonei (PMP) is a rare malignant disease mainly originated from appendiceal mucinous tumors, which often produced mucinous and gelatinous masses [1]. The clinical presentation of PMP varies widely and depends on the disease course; however, due to its low incidence rate, the diagnosis is always challenging [2]. Cytoreductive surgery (CRS) in combination with hyperthermic intraperitoneal chemotherapy (HIPEC) is the active treatment for PMP [3]; since then, the prognosis of PMP patients has been greatly improved, with both progression-free survival (*PFS*) and overall survival (*OS*) being significantly prolonged.

Completeness of cytoreduction is one of the most important prognostic factors for PMP [4–6], as affirmed by our previous retrospective cohort study [7]. The completeness of cytoreduction depends primarily on the surgical skills of the surgeon [8] and the tumor load of PMP. The peritoneal cancer index (PCI) is employed to estimate the tumor load resulting from peritoneal metastasis, which exhibits a negative correlation with the chances of cytoreduction [9]. Consequently, there is an urgent need to identify PCI cutoff point for predicting surgical resectability in PMP patients.

In clinical practice, the PCI cutoff point differs between low-grade and high-grade PMP patients, as low-grade PMP patients are more likely to achieve complete CRS than the high-grade subjects when the tumor load is equivalent. During the surgical removal of tumors, it is technically challenging to resect massive tumors with wide intra-abdominal involvement, including the hepatic hilum (region 2) and small intestine (regions 9–12) [10, 11]. A former study confirmed that selected PCI regions (region 2 + 9–12), corresponding to the small intestine and hepatoduodenal ligament, were more predictive of complete resection in advanced epithelial ovarian cancer [12]. The present study aimed to determine the optimal cutoff point for total and selected PCI to predict surgical resectability for both low-grade and high-grade PMP patients, which could contribute to preoperative patient selection and/or information.

## Materials and methods

### Patients

**Fig. 1:**
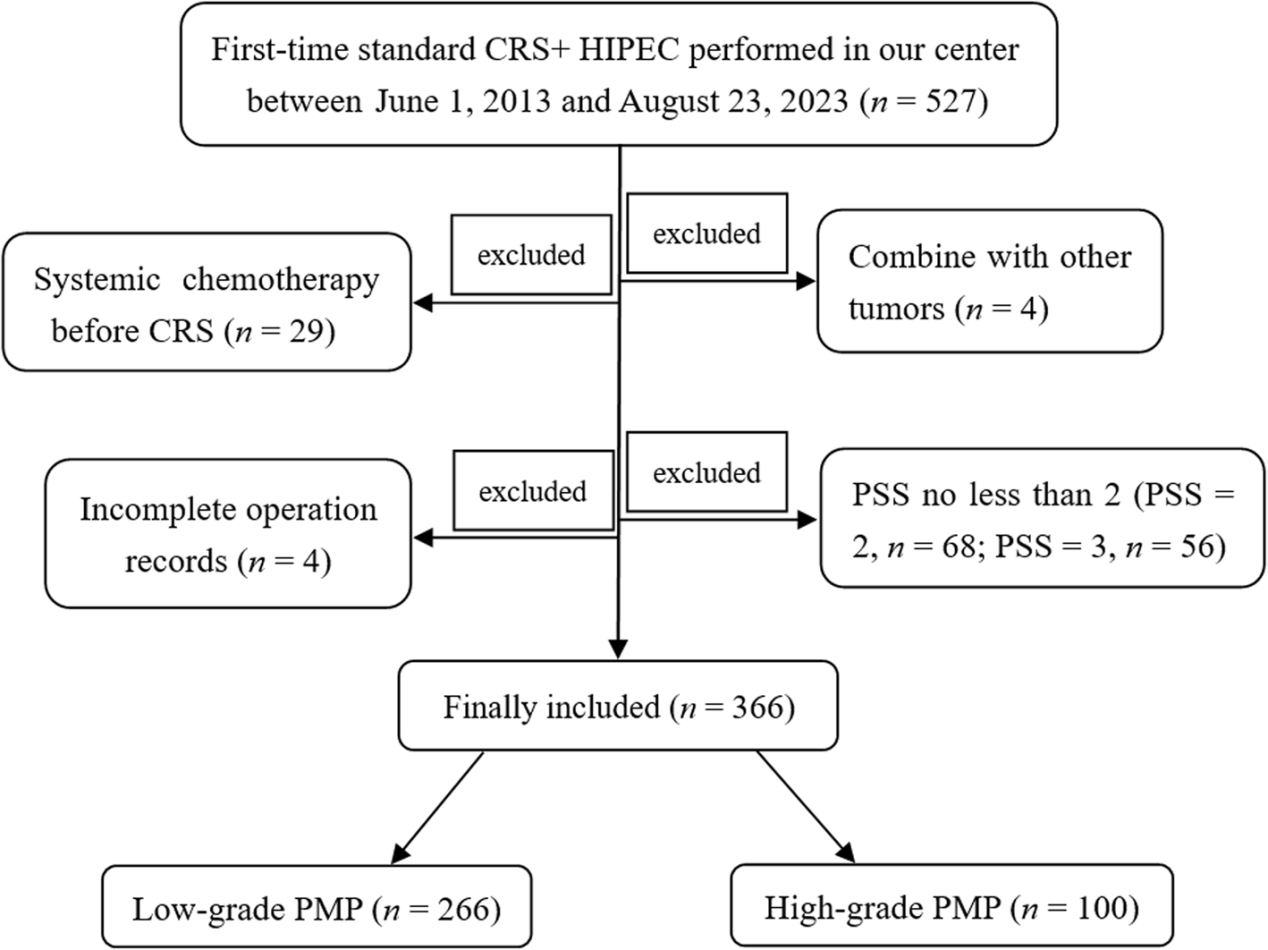
Study schematic. A total of 527 PMP patients whose first-time standard CRS + HIPEC performed in our center were retrieved between June 1, 2013, and August 23, 2023. Patient combined with other tumors (*n* = 4), received systemic chemotherapy before CRS (*n* = 29), incomplete surgery record (*n* = 4), and with *PSS* ≥ 2 (*PSS* = 2, *n* = 68; *PSS* = 3, *n* = 56) were all excluded. Finally, 366 PMP patients were included, including 266 low-grade and 100 high-grade subjects. PMP, pseudomyxoma peritonei; CRS, cytoreductive surgery; HIPEC, hyperthermic intraperitoneal chemotherapy; PSS, prior surgical score

The Institutional Review Board (IRB) of Aerospace Center Hospital approved the present study (*no*. 20200113-LCYJ-01). All patients provided signed informed consent before the operation and consented to follow-up post-surgery. Only the PMP patients whose first-time standard CRS + HIPEC performed in our center met the inclusion criteria, resulting in a total of 527 subjects, acquired between June 1, 2013, and August 23, 2023. The exclusion criteria included the following aspects: (1) Combined with other tumors (one with breast cancer, one with stomach cancer, one with lung cancer, and one with oral cancer, *n* = 4), (2) received systemic chemotherapy before CRS (*n* = 29), (3) incomplete surgery record (*n* = 4), and (4) prior surgical score (PSS) ≥ 2 (*PSS* = 2, *n* = 68; *PSS* = 3, *n* = 56). Finally, a total of 366 PMP patients were included in present study, comprising 266 low-grade and 100 high-grade subjects (Fig. 1).

### PCI calculation and CRS

The PCI was assessed during laparotomy by dividing the abdomen and pelvis into 13 regions (0–12) and assigning a lesion size score (0–3) to each region [3]. The sum of all 13 individual lesion size scores, defined as total PCI, ranged from 0 to 39 [13]. The completeness of cytoreduction (CC) was divided into four grades by evaluating the size of residual tumor tissue, which were CC-0 (no residual tumor), CC-1 (residual tumor deposits from 0 to 2.5 mm), CC-2 (residual tumor deposits from 2.5 mm to 2.5 cm), and CC-3 (residual tumor deposits more than 2.5 cm), respectively. A CC-0/1 score indicated patients who underwent complete CRS, while a CC-2/3 score indicated incomplete cytoreduction, also defined as major tumor debulking (MTD) [14]. The selected PCI scores for region (2 + 9 to 12) will be calculated simultaneously, and their value in predicting surgical resectability will be evaluated separately. 

HIPEC was performed after CRS. Following the temporary closure of the abdominal skin, mitomycin (20 mg/m^2^) or cisplatin (80–100 mg/m^2^) was warmed to a temperature of 41–42 °C for 60–90 min. Two experienced pathologists interpreted all resected specimens according to the Peritoneal Surface Oncology Group (PSOGI) classification criteria [5].

### Statistical analysis

All statistical analyses were performed using *SPSS* (version 16.0; IBM Corporation, Armonk, NY, USA), *GraphPad prism* (version 8.0; GraphPad Software), and *MedCalc* (version 15.2.2; MedCalc Software, Flanders, Belgium). *Chi-square* test was employed to compare rates between groups. The comparison of quantities between groups was performed using either independent sample *T*-test or *Mann-Whitney U*-test, as appropriate.

The discriminative ability between total and selected PCI for predicting surgical resectability of PMP was compared by AUC of receiver operating characteristic (ROC) curves, utilizing a nonparametric approach developed by DeLong et al. [15]. The optimal total and selected PCI cutoff points were then determined. An AUC of 0.9–1.0 indicated an excellent test, 0.8–0.9 indicated a good test, 0.7–0.8 indicated a fair test, 0.6–0.7 indicated a poor test, while 0.5–0.6 indicated a failed test [16].

Once the optimal cutoff point was determined, the low-grade and high-grade PMP patients were further divided into low and high PCI subgroups, respectively. Subsequently, the *Kaplan–Meier* survival analysis was performed between the aforementioned subgroups for both low-grade and high-grade PMP subjects. A two-sided *p*-value less than 0.05 was considered to be statistically significant.

## Results

**Fig. 2:**
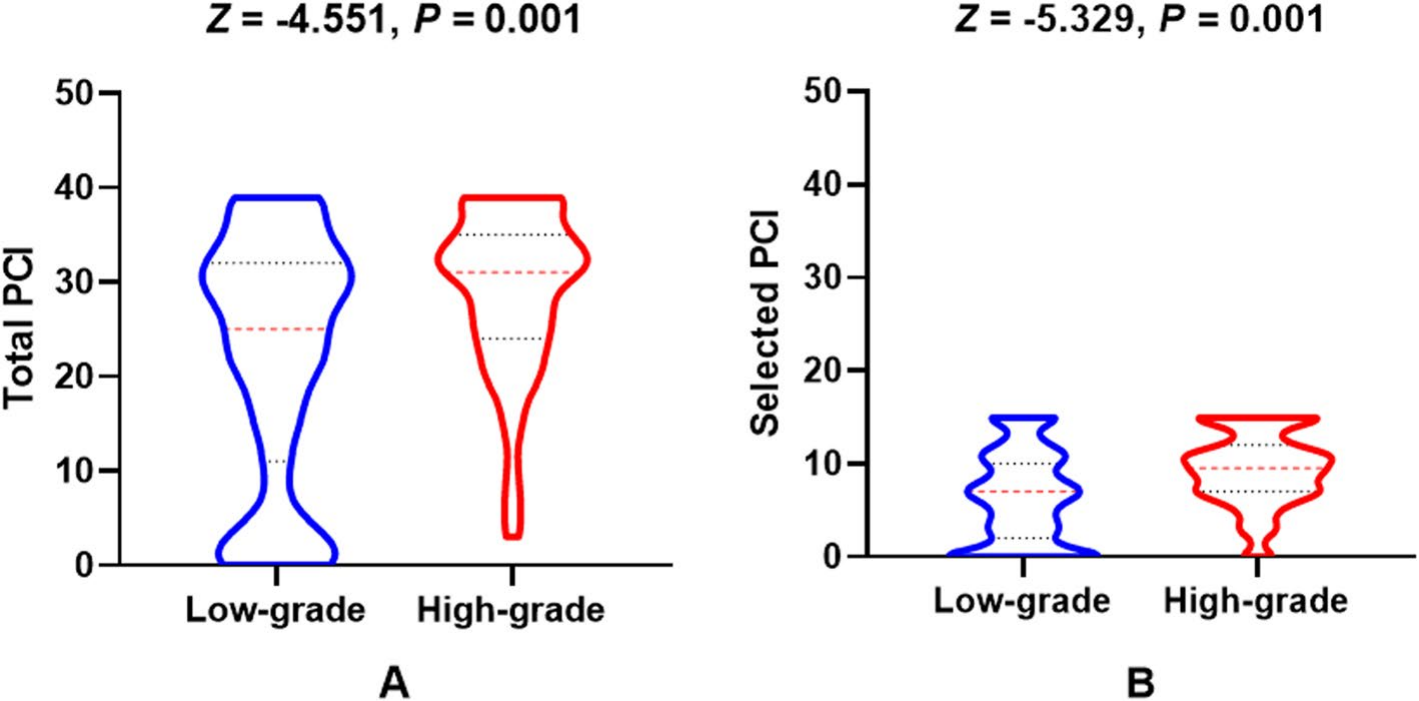
Total and selected PCI levels between low-grade and high-grade PMP patients (all *p* = 0.001)

In low-grade PMP patients, 119 underwent complete CRS and 147 underwent MTD, while in high-grade PMP patients, 24 underwent complete CRS and 76 underwent MTD (*χ*^2^ = 13.128, *p* = 0.001). The total PCI level between low-grade and high-grade PMP patients was 25 (11, 32) *vs.* 31 (24, 35), *Z* =  − 4.551, *p* = 0.001. Similarly, the selected PCI level between low-grade and high-grade PMP patients was 7 (2, 10) *vs.* 10 (7, 12), *Z* =  − 5.329, *p* = 0.001 (Fig. 2).

### Low-grade PMP patients

**Fig. 3:**
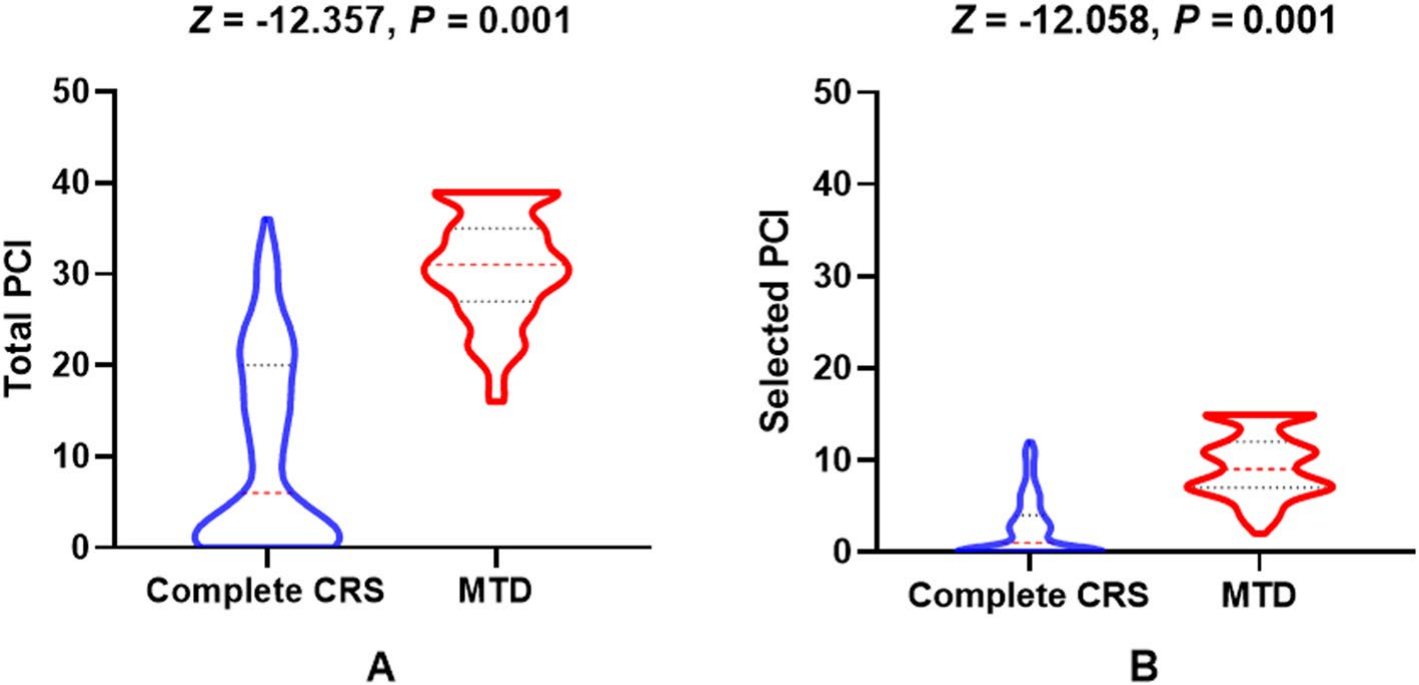
Total and selected PCI levels between complete CRS and MTD in low-grade PMP patients (all *p* = 0.001)

For the low-grade PMP patients, 47 males and 72 females were in the complete CRS subgroup, while 94 males and 53 females were in the MTD subgroup (*χ*^2^ = 15.782, *p* = 0.001). The mean age between the complete CRS and MTD group was 54.3 ± 11.5 *vs.* 59.0 ± 10.4 years, *t* =  − 3.455, *p* = 0.001. The *median* total PCI differed between the complete CRS and MTD subgroups, with values of 6 (1, 20) *vs.* 31 (27, 35), *Z* =  − 12.357, *p* = 0.001. Similarly, the *median* selected PCI differed between the complete CRS and MTD subgroups, with values of 1 (0, 4) *vs.* 9 (7, 12), *Z* =  − 12.058, *p* = 0.001 (Fig. 3). The detail clinicopathological characteristics are shown in Table 1. Tumor histological sources included appendix (*n* = 260), ovarian (*n* = 3), colorectal (*n* = 2), and gallbladder (*n* = 1). There was one patient with acellular mucin and 265 with disseminated peritoneal adenomucinosis (DPAM).

**Table 1:** Clinicopathological characteristics between complete CRS and MTD subgroups of low-grade PMP patients

	Complete CRS group (*n* = 119)	MTD group (*n* = 147)	*p*-value
Sex (male/female)	47/72	94/53	0.001
Age (years)	54.3 ± 11.5	59.0 ± 10.4	0.001
Hospital time (days)	21.0 ± 8.0	24.4 ± 6.1	0.001
Barthel Index score	100 (100, 100)	100 (95, 100)	0.014
Operation time (hours)	7.2 ± 2.5	7.9 ± 2.0	0.014
Total PCI	6 (1, 20)	31 (27, 35)	0.001
Selected PCI (2 + 9 to 12)	1 (0, 4)	9 (7, 12)	0.001

*CRS* cytoreductive surgery, *MTD* maximal tumor debulking, *PMP* pseudomyxoma peritonei, *PCI* peritoneal cancer index

**Fig. 4:**
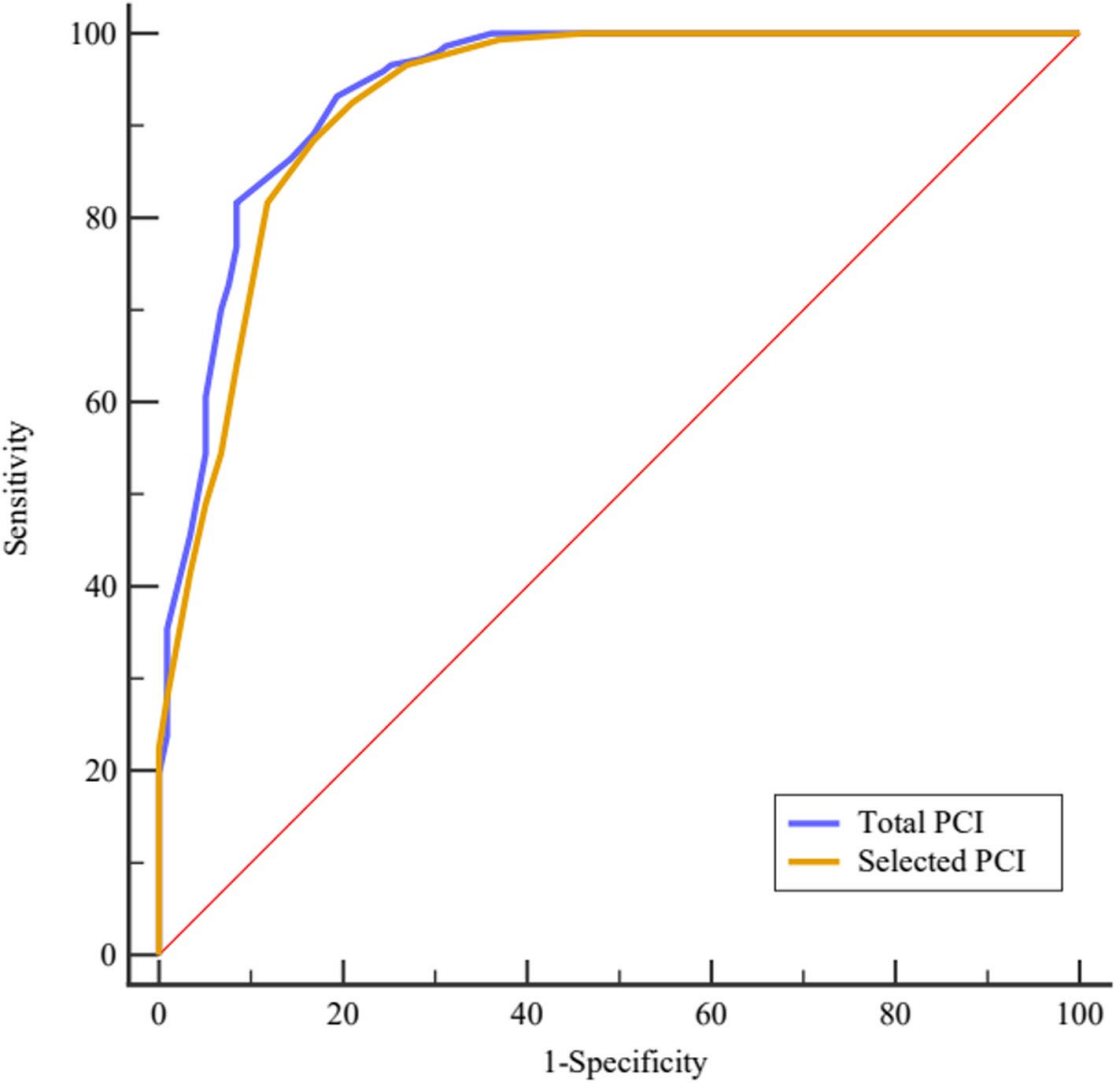
The ROC curves of total PCI and selected PCI to predict surgical resectability for low-grade PMP patients

Surgical resection was regarded as the gold standard; the ROC-AUC of total PCI in predicting surgical resectability was 0.940 [95% *CI*: 0.904–0.965], with an optimal cutoff point of 21. The corresponding sensitivity was 93.20%, and specificity was 80.67%, resulting in a Youden index of 0.739. The ROC-AUC of selected PCI regions (2 + 9 to 12) to determine surgical resectability for PMP was 0.927 (95% *CI*: 0.889–0.955), with the optimal cutoff point of 5. The sensitivity was 88.44%, and specificity was 83.19%, resulting in a Youden index of 0.716. The discriminative ability of total and selected PCI in predicting surgical resectability was compared by the method of DeLong et al., and the AUC difference did not show a statistical difference (*Z* = 1.462, *p* = 0.144). Details are shown in Table 2 and Fig. 4.

**Table 2:** Details of total and selected PCI for predicting surgical resectability of low-grade PMP patients

	AUC (95% *CI*)	Cutoff point	Sensitivity (%)	Specificity (%)	Youden index
Total PCI	0.940 (0.904–0.965)	21	93.20	80.67	0.739
Selected PCI (2 + 9 to 12)	0.927 (0.889–0.955)	5	88.44	83.19	0.716

*PCI* peritoneal cancer index, *PMP* pseudomyxoma peritonei

**Fig. 5:**
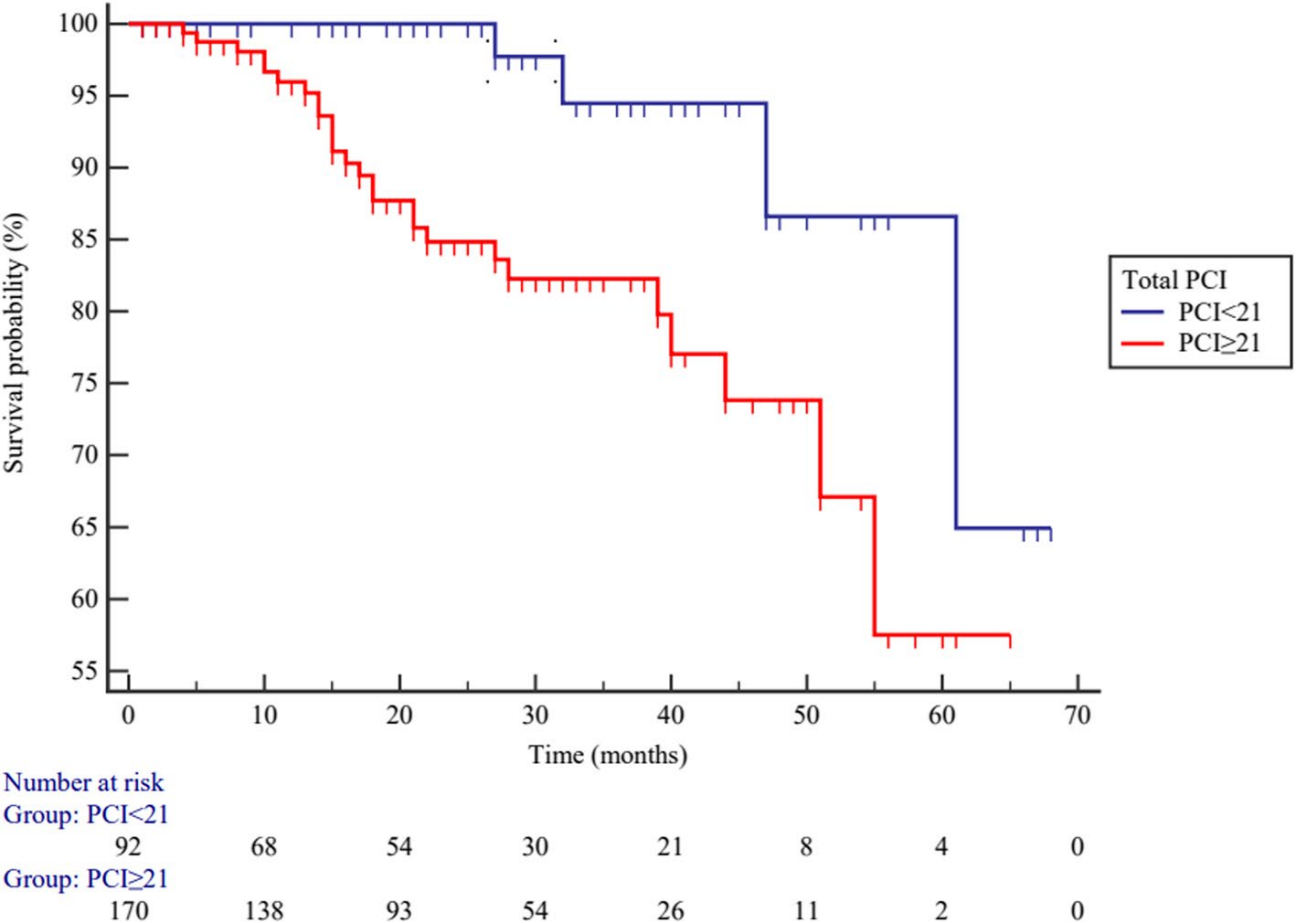
The overall survival between higher (*PCI* ≥ 21) and lower (*PCI* < 21) total PCI subgroups in low-grade PMP patients (*p* = 0.005)

**Fig. 6:**
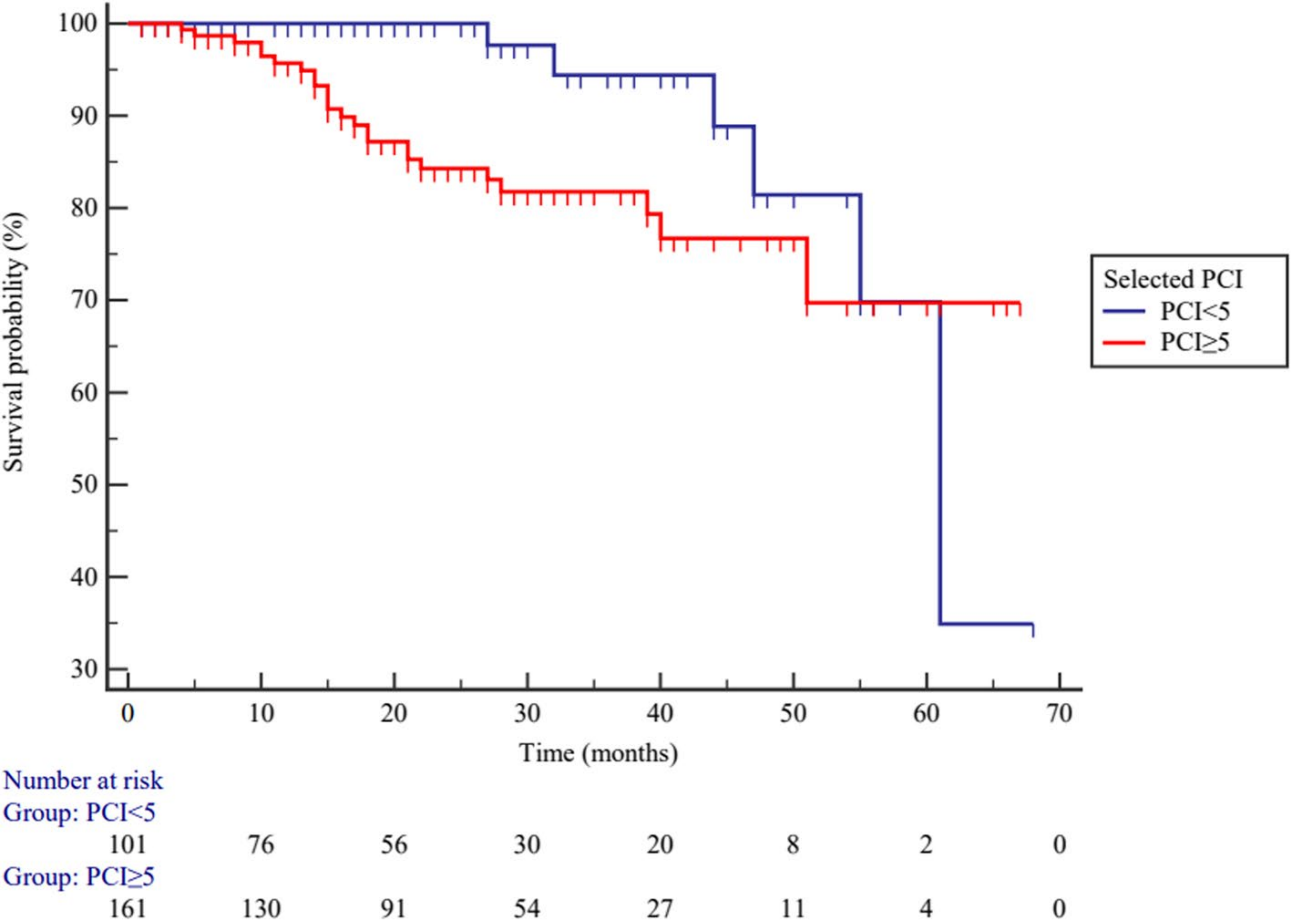
The overall survival between higher (*PCI* ≥ 5) and lower (*PCI* < 5) selected PCI subgroups in low-grade PMP patients (*p* = 0.035)

According to the optimal cutoff point of total PCI, the low-grade PMP patients were divided into higher (*PCI* ≥ 21) and lower (*PCI* < 21) PCI subgroups. Elevated total PCI levels are associated with poor overall survival (*OS*) in patients with PMP, *HR* = 2.939 (95% *CI*: 1.397–6.182), *p* = 0.005 (Fig. 5). Low-grade PMP patients were also divided into higher (*PCI* ≥ 5) and lower (*PCI* < 5) PCI subgroups according to the selected PCI; the elevated selected PCI levels are also associated with poor *OS* in patients with PMP, *HR* = 2.210 (95% *CI*: 1.058–4.618), *p* = 0.035 (Fig. 6).

### High-grade PMP patients

**Fig. 7:**
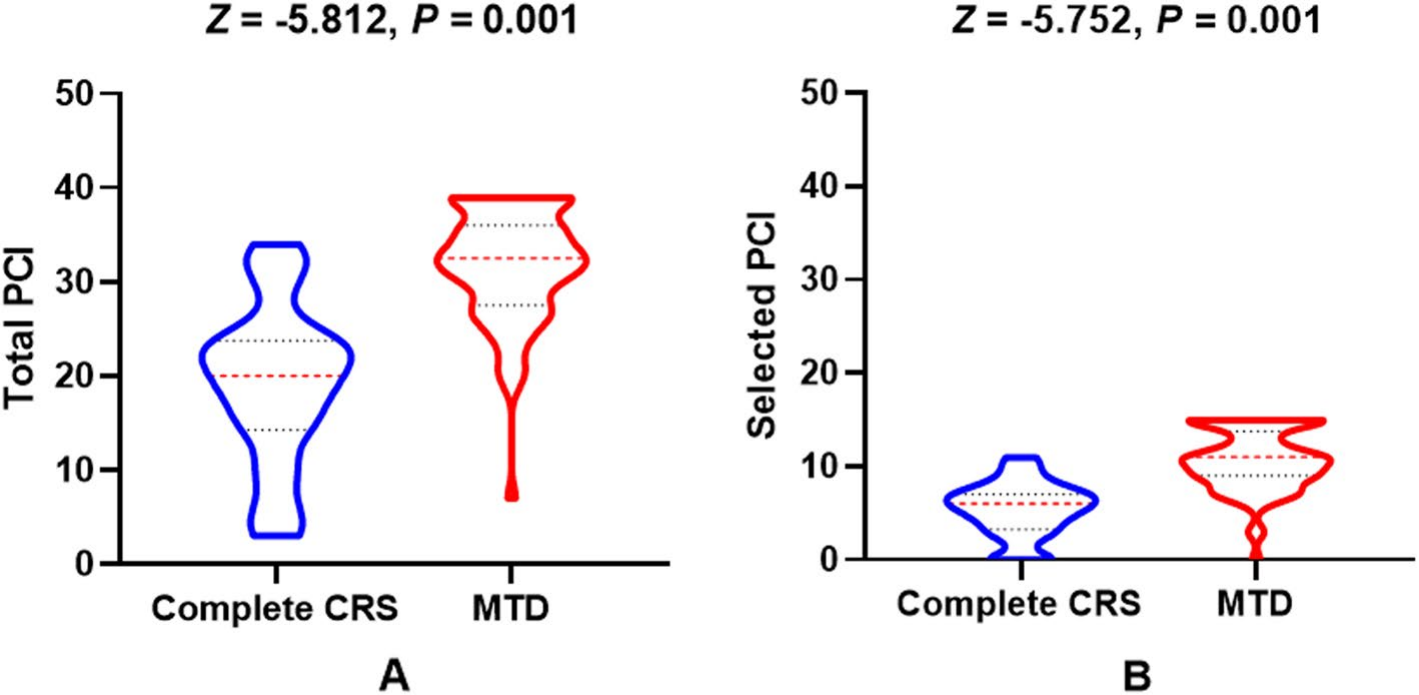
The total and selected PCI levels between complete CRS and MTD in high-grade PMP patients (all *p* = 0.001)

For the high-grade PMP patients, 10 males and 14 females were in the complete CRS subgroup, while 46 males and 30 females were in the MTD subgroup (*χ*^2^ = 2.633, *p* = 0.105). The *median* total PCI between complete CRS and MTD subgroups was 20 (15, 24) *vs.* 33 (28, 36), *Z* =  − 5.812, *p* = 0.001. The *median* selected PCI between complete CRS and MTD subgroups was 6 (4, 7) *vs.* 11 (9, 14), *Z* =  − 5.752, *p* = 0.001 (Fig. 7). The detailed clinicopathological characteristics are shown in Table 3. Tumor histological sources included appendix (*n* = 87), colorectal (*n* = 8), ovarian (*n* = 2), urachus (*n* = 1), gallbladder (*n* = 1), and small intestinal (*n* = 1). There were 76 patients with peritoneal mucinous carcinomatosis (PMCA) and 24 with peritoneal mucinous carcinomatosis with signet ring cells (PMCA-S).

**Table 3:** Clinicopathological characteristics between complete CRS and MTD subgroups of high-grade PMP patients

	Complete CRS group (*n* = 24)	MTD group (*n* = 76)	*p*-value
Sex (male/female)	10/14	46/30	0.105
Age (years)	57.0 ± 10.3	58.2 ± 11.4	0.640
Hospital time (days)	26.5 ± 8.7	26.3 ± 7.9	0.944
Barthel Index score	100 (95, 100)	95 (90, 100)	0.055
Operation time (hours)	8.8 ± 2.0	7.8 ± 2.2	0.038
Total PCI	20 (15, 24)	33 (28, 36)	0.001
Selected PCI (2 + 9 to 12)	6 (4, 7)	11 (9, 14)	0.001

*CRS* cytoreductive surgery, *MTD* maximal tumor debulking, *PMP* pseudomyxoma peritonei, *PCI* peritoneal cancer index

**Fig. 8:**
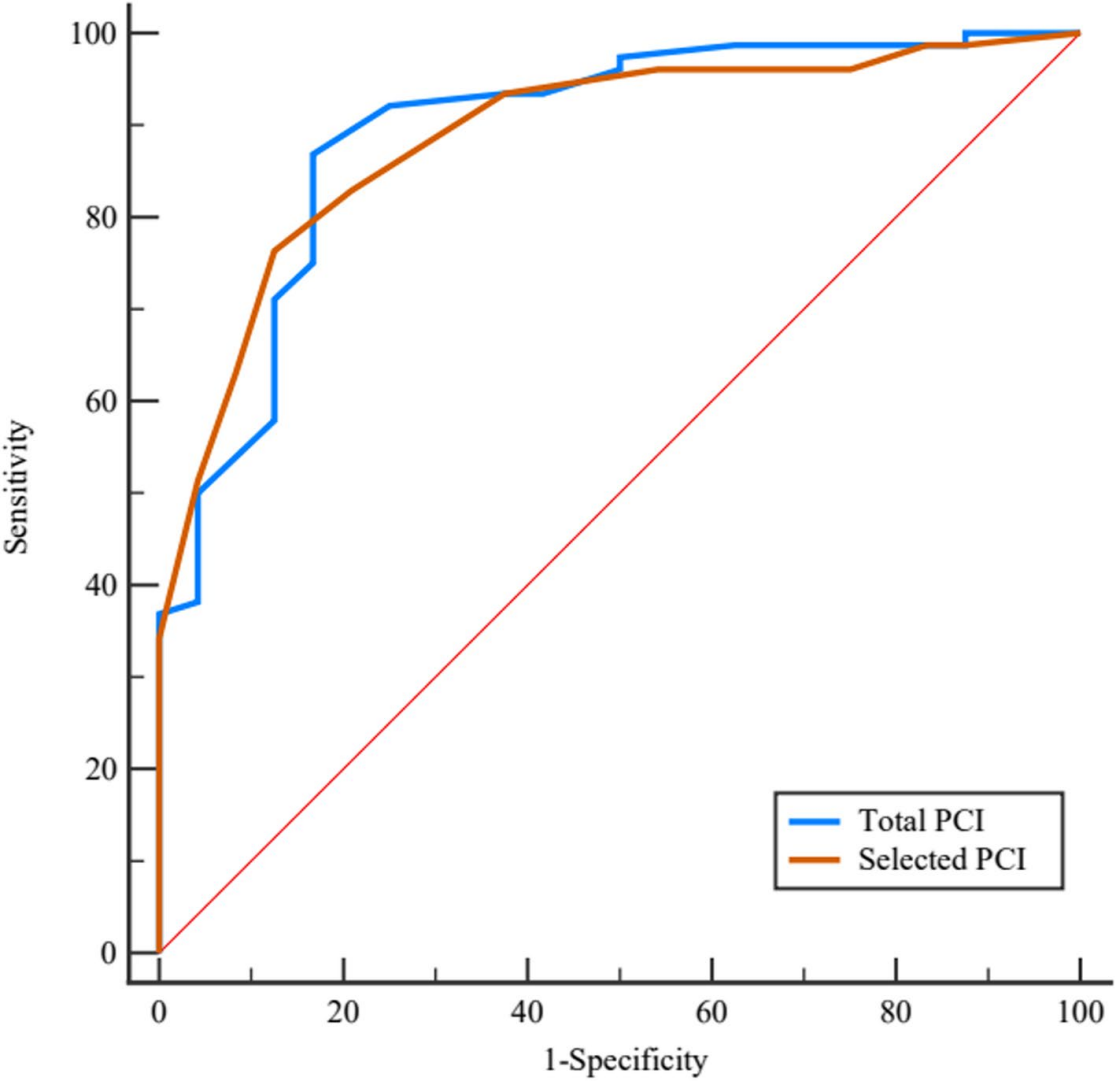
The ROC curves of total PCI and selected PCI for predicting surgical resectability in high-grade PMP patients

The ROC-AUC of total PCI in predicting surgical resectability for high-grade PMP was 0.894 (95% *CI*: 0.816–0.946), with an optimal cutoff point of 25. The corresponding sensitivity was 86.84%, and specificity was 83.33%, resulting in a Youden index of 0.702. Similarly, the ROC-AUC of selected PCI regions was 0.888 (95% *CI*: 0.810–0.943), with an optimal cutoff point of 8. The corresponding sensitivity was 76.32%, and specificity was 87.50%, resulting in a Youden index of 0.638. The AUC difference between total and selected PCI in predicting surgical resectability did not reach statistical difference (*Z* = 0.245, *p* = 0.806). Details are shown in Table 4 and Fig. 8.

**Table 4:** Details of total and selected PCI for predicting surgical degree of high-grade PMP patients

	AUC (95% *CI*)	Cutoff point	Sensitivity (%)	Specificity (%)	Youden index
Total PCI	0.894 (0.816–0.946)	25	86.84	83.33	0.702
Selected PCI (2 + 9 to 12)	0.888 (0.810–0.943)	8	76.32	87.50	0.638

*PCI* peritoneal cancer index, *PMP* pseudomyxoma peritonei

**Fig. 9:**
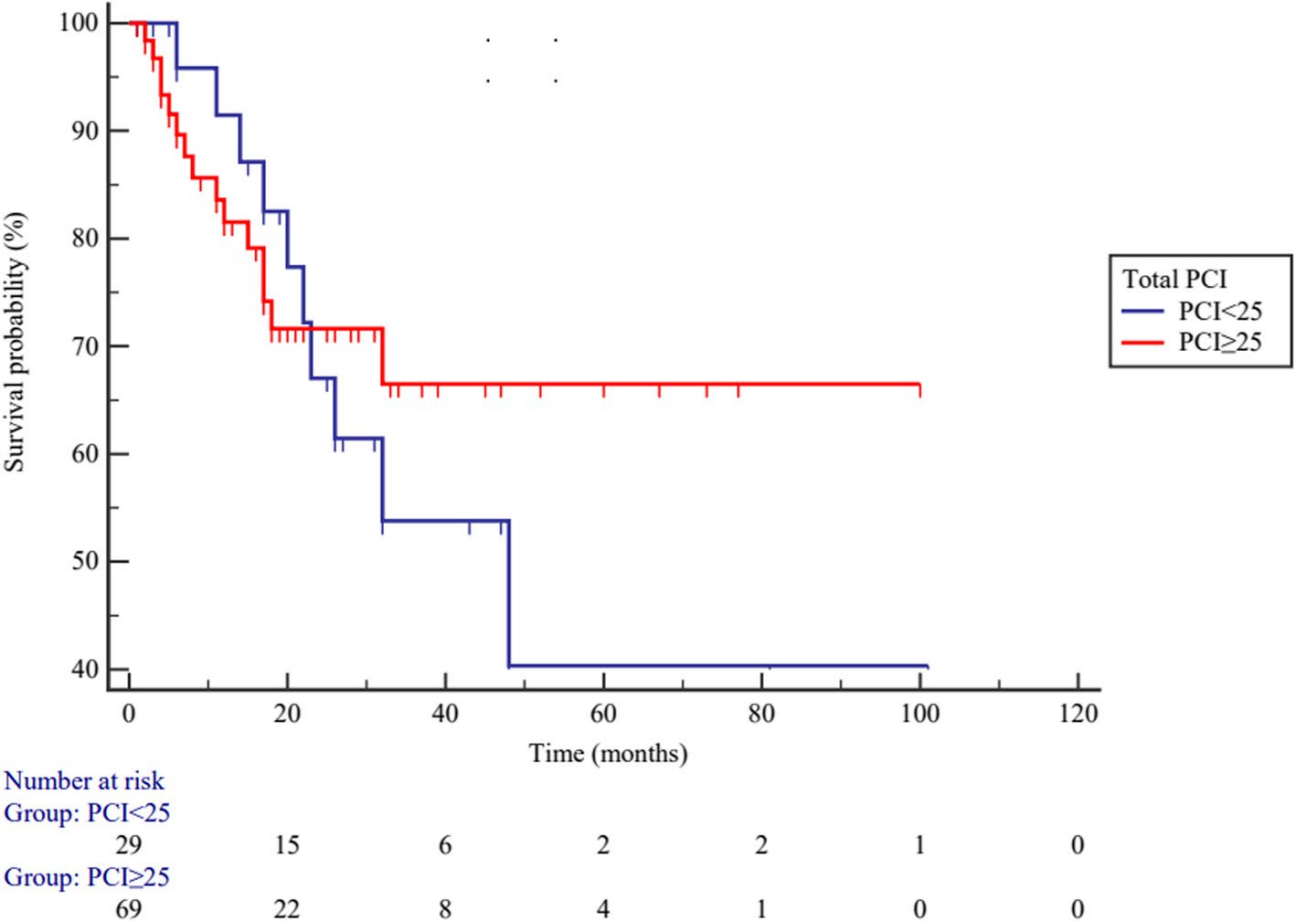
The overall survival between higher (*PCI* ≥ 25) and lower (*PCI* < 25) total PCI subgroups in high-grade PMP patients (*p* = 0.647)

**Fig. 10:**
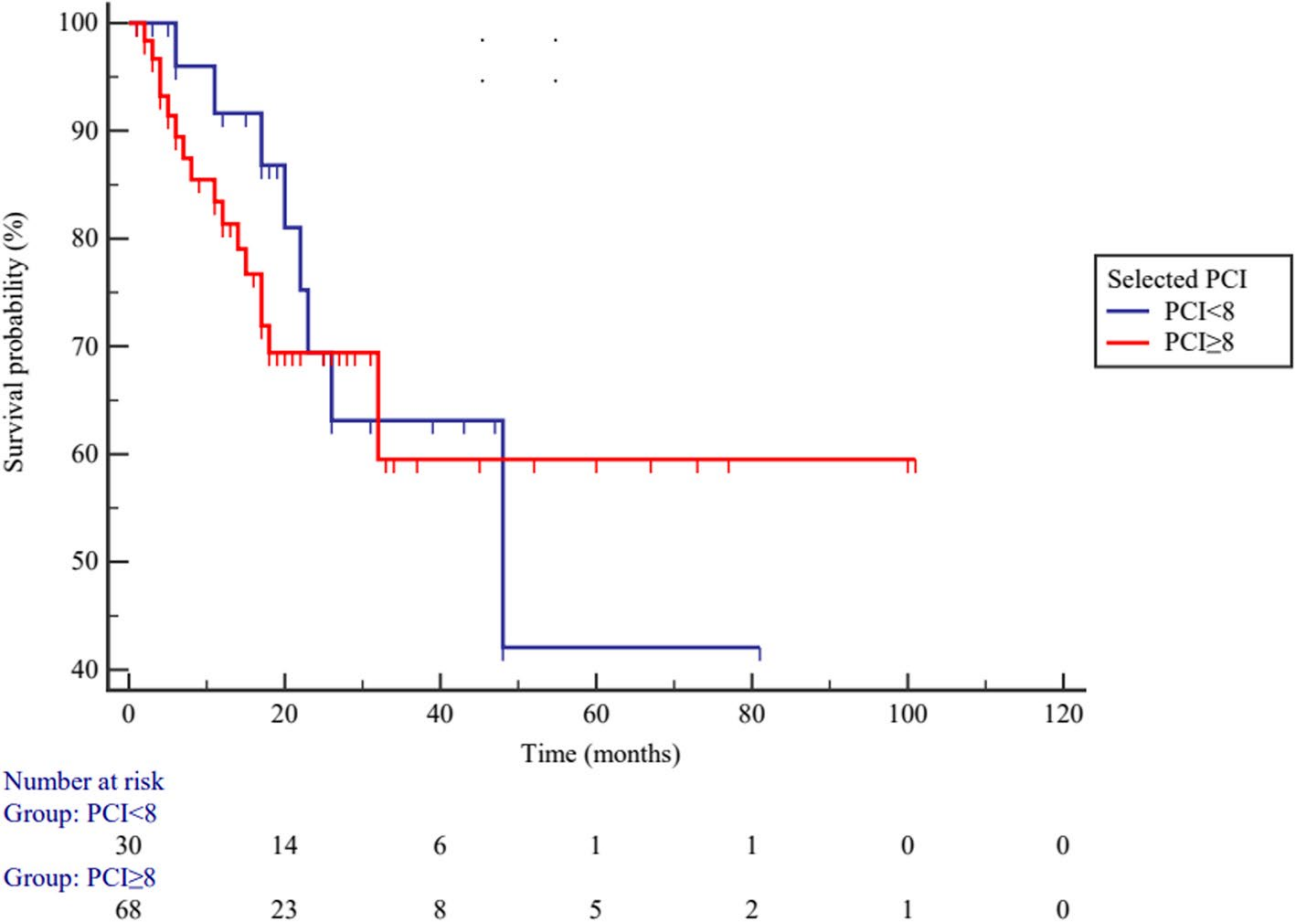
The overall survival between higher (*PCI* ≥ 8) and lower (*PCI* < 8) selected PCI subgroups in high-grade PMP patients (*p* = 0.746)

According to optimal cutoff point of total PCI, high-grade PMP patients were divided into higher (*PCI* ≥ 25) and lower (*PCI* < 25) PCI subgroups. Elevated total PCI levels are not associated with poor *OS* in patients with PMP, *HR* = 0.825 (95% *CI*: 0.361–1.883), *p* = 0.647 (Fig. 9). High-grade PMP patients were also divided into higher (*PCI* ≥ 8) and lower (*PCI* < 8) PCI subgroups according to the selected PCI, and elevated selected PCI levels are also not associated with poor *OS* in patients with PMP, *HR* = 1.147 (95% *CI*: 0.501–2.623), *p* = 0.746 (Fig. 10).

## Discussions

The present study identified a significantly higher PCI level in high-grade PMP patients compared to low-grade subjects. The discriminative ability of both total and selected PCI to predict surgical resectability for low-grade PMP patients demonstrated excellent performance, with optimal cutoff points of 21 and 5, respectively. Similarly, for high-grade PMP patients, both total and selected PCI exhibited good performance in predict surgical resectability, with optimal cutoff points of 25 and 8, respectively.

Completeness of cytoreduction emerged as the most critical prognostic factor for PMP [5, 6, 17], with PCI is negatively correlated with the ability to achieve a complete CRS [18]. Patient selection can be challenging in PMP cases with very high PCI [19], as postoperative morbidity and mortality associated with CRS should be taken into account for such an extensive disease. Therefore, various studies have established PCI cutoff point to assist in preoperative patient selection for PMP.

Reviewing previous studies [3, 9, 18, 20], we observed variations in PCI cutoff point for predicting resectability. We speculate that these differences stem mainly from variations in the included populations. For instance, Votanopoulos K. I. et al. found that 21% of high-grade PMP patients achieved a complete CRS when the PCI was ≥ 21. In our research, among 85 high-grade PMP patients with *PCI* ≥ 21, only 14% (12/85) reached complete CRS. We speculate that this difference is mainly attributed to the different tumor loads of the included population, with our research showing a higher mean PCI of 28.8 compared to 14.8 in the former study. Additionally, the present study found a higher PCI level in high-grade PMP compared to low-grade PMP, which also result in the higher PCI cutoff point in high-grade PMP patients. This may be related to the biological behavior of the malignancy, since patients with a high PCI are likely to have more aggressive disease than those with a low PCI.

In 2002, Sugarbaker proposed that total gastrectomy with a temporary diverting jejunostomy could be employed to facilitate complete cytoreduction in patients with advanced PMP syndrome [21]. By 2020, Kitai T. et al. [3] reported that 69.0% (20/29) of extensive PMP patients underwent complete CRS, with all 20 patients who underwent gastrectomy based on the aforementioned surgical concept. However, this approach resulted in a high postoperative complication rate. Benhaim et al. reported that 54% (54/100) of extensive PMP patients achieved complete CRS. They concluded that technical progress contributed to increasing the complete CRS rate of PMP patients from 25 to 71% [10]. Due to the potential adverse impact of gastrectomy on daily life after surgery [22], our center prioritizes stomach-sparing surgery whenever possible, emphasizing that all treatments for PMP patients aim not only to keep them alive but in life [23].

Overall, the determination of the optimal PCI cutoff point may be affected by the three key factors: the tumor load of PMP patients, the surgical approach, and the surgeon’s expertise. To establish a standardized PCI value for predicting surgical outcomes, our preliminary recommendation is to conduct multicenter research on a global scale. Moreover, it is crucial for all centers to be recognized as professional PMP centers.

The present study found that the discriminative ability of selected PCI and total PCI in predicting surgical resectability was similar for both low-grade and high-grade PMP patients. To our knowledge, this is the first attempt to compare the selected PCI with total PCI for PMP patients. The total PCI has certain limitations, which can only be assessed after complete lysis of all adhesions [24], with complete abdominal examination [10]; when confronting high tumor load patients, it is always time-demanding and cannot be obtained by laparoscopy [12]. Although present study did not find a statistical difference between selected and total PCI in predicting complete cytoreduction for PMP patients, the selected PCI can be easily applied and time-saving to predict the surgical outcome for PMP patients. Hence, the selected PCI demonstrates better practicality in clinical practice.

According to the PCI cutoff point, the subgroups for both total and selected PCI of low-grade PMP patients exhibited different prognoses, while in high-grade PMP patients, no difference were observed. Through statistical analysis, it was found that only 25 high-grade PMP patients reached endpoint events. When combined with the *Kaplan–Meier* plots, the curves of the two subgroups showed intersection point. We consider that if the effective sample size of high-grade PMP patients is further increased, the PCI may also have predictive value.

There were several limitations in the present study. Firstly, PMP appears to be more common in women, who often present with rapidly enlarging ovarian masses [25]. Nevertheless, the gender ratio (male/female) between included and excluded PMP patients in our study was 197/169 and 47/114 (*χ*^2^ = 27.287, *p* = 0.001), indicating a certain selection bias in the present research. Secondly, only 39% (143/366) of PMP patients underwent complete resection, a rate lower than that reported in foreign PMP centers. Thirdly, all participants were consecutively included over a 10-year period, and the complete cytoreduction rate increased during that time. Lastly, PCI can only be acquired during surgery, thus not contribute to preoperative patient selection. Fortunately, computer tomography (CT) determined PCI has been employed to accurately calculate PCI preoperatively by the experienced radiologists [26]. In the future, there is a tremendous need to establish a prediction model to preoperatively calculate PCI.

In conclusion, the PCI cutoff point demonstrated good performance in predicting surgical resectability for both low-grade and high-grade PMP patients. The discriminative ability of total and selected PCI was similar; nevertheless, selected PCI was simpler and time-saving in clinical practice. In the future, new imaging techniques or predictive models may be developed to better predict PCI preoperatively, which might assist in confirming whether complete surgical resection can be achieved.

## Data Availability

No datasets were generated or analysed during the current study.

## Abbreviations

PMP: Pseudomyxoma peritonei
CRS: Cytoreductive surgery
HIPEC: Hyperthermic intraperitoneal chemotherapy
PFS: Progression-free survival
OS: Overall survival
PCI: Peritoneal cancer index
PSS: Prior surgical score
CC: Completeness of cytoreduction
MTD: Maximum tumor debulking
PSOGI: Peritoneal Surface Oncology Group
ROC: Receiver operating characteristic
DPAM: Disseminated peritoneal adenomucinosis
PMCA: Peritoneal mucinous carcinomatosis
PMCA-S: Peritoneal mucinous carcinomatosis with signet ring cells
CT: Computer tomography
